# Is Orthodontic Treatment a Risk Factor of Cervical Root Resorption? A Systematic Review

**DOI:** 10.1055/s-0041-1742127

**Published:** 2022-02-23

**Authors:** Khaled Khalaf, Shahd Mustafa, Tamara Maarouf

**Affiliations:** 1 Department of Preventive and Restorative Dentistry, College of Dental Medicine, University of Sharjah, Sharjah, United Arab Emirates; 2 Institute of Dentistry, School of Medicine, Medical Sciences & Nutrition, University of Aberdeen, Aberdeen, United Kingdom

**Keywords:** cervical root resorption, orthodontic treatment, tooth movement, systematic review

## Abstract

Cervical root resorption is an uncommon, aggressive form of external resorption that occurs on the root surface of a permanent tooth and presents clinically as a characteristic pinkish discoloration of the tooth. The cause of cervical root resorption is poorly understood, and it has been suggested that orthodontic treatment may play a role in causing this pathological condition. The aim of this systematic review was to investigate whether orthodontic treatment could be considered as a risk factor of cervical root resorption in patients who had undergone fixed appliances therapy. A comprehensive electronic and manual search was conducted in four databases and six journals without any limitations on year of publication. A customized data extraction form was used to retrieve relevant information from each eligible study. Risk of bias was assessed using the Cochrane risk of bias tool (RoB 2) and the risk of bias in nonrandomized studies of interventions tool (ROBINS-I). The quality of evidence was assessed using the grading of recommendations, assessment, development, and evaluation (GRADE) approach. Two randomized clinical trials (RCTs) and one cohort study were included in the review. Risk of bias was assessed as high for both RCTs and critical for the cohort study. Conflicting results were reported by the studies included in this review. Both RCTs reported significant differences between orthodontically moved teeth and teeth that were not moved, while the cohort study reported a lack of association between fixed orthodontic treatment and cervical root resorption. Quality of evidence provided by this review was judged to be of very low quality. Orthodontic treatment may have potential in inducing cervical root resorption; however, due to the high risk of bias of the included studies and their conflicting findings, better-quality studies are needed to make definitive conclusions.

## Introduction

Root resorption is a condition that occurs when there is a partial loss of cementum and dentin. Generally, root resorption can arise physiologically during exfoliation of the deciduous teeth, or as a pathological inflammatory process caused by injury to the root surfaces of permanent teeth. 1 If this inflammatory resorption is seen on the inner wall of the root canal, it is known as internal resorption, whereas if it was on the external surface of the root, it is called external resorption. 2 External root resorption can be further subclassified as external replacement resorption, surface resorption, external inflammatory resorption, transient apical breakdown, and external cervical resorption. 3 External cervical resorption can be referred to by many clinical terms including invasive cervical resorption, peripheral cervical resorption, subepithelial external root resorption, and cervical root resorption. 4 5 6 7 Due to its many names, this type of external resorption will be referred to as cervical root resorption throughout this study to avoid any confusion.

Cervical root resorption is an uncommon and aggressive form of external resorption which occurs on a permanent tooth's root surface below the coronal surface of the supporting alveolar process and the epithelial attachment. After the loss of tooth structure, the resorbed area is then replaced by tissue that is highly vascular in nature which can be seen through the thin residual enamel. This vascular tissue presents clinically as a characteristic pinkish discoloration of the tooth. 8 Cervical root resorption is a multifactorial disease with a poorly understood cause. It is presumed that physical or chemical damage to the precementum increases the prevalence of cervical root resorption. Several predisposing factors such as trauma, periodontal treatment, internal bleaching, and orthodontic treatment have been reported to have a strong association with cervical root resorption. 9 10 11 There are few studies that have reported the occurrence of cervical root resorption in orthodontic patients, possibly due to the fact that clinical manifestation of cervical root resorption often takes a lot of time to develop after the initial stimulus. 8 Heithersay 12 reported that orthodontic treatment was the most common sole predisposing factor for 21.2% of patients and 24.1% of teeth with cervical root resorption. However, Irinakis et al 13 stated that only 3.9% of patients with cervical root resorption had undergone orthodontic treatment.

Therefore, the aim of this systematic review was to investigate whether orthodontic treatment could be considered as a risk factor of cervical root resorption in patients who had undergone fixed appliances therapy.

## Methods

### Protocol and Registration

This systematic review was conducted in accordance with the preferred reporting items for evaluation of articles in systematic reviews and meta-analysis (preferred reporting items for systematic review and meta-analysis [PRISMA]) guidelines. 14 The protocol of the review was also registered in the international prospective register of systematic reviews (PROSPERO) under the registration number CRD42021256278.

### Selection of Studies and Eligibility Criteria

Prior to starting the search, the population, intervention, control, outcomes, and study design (PICOS) were outlined to formulate a structured design as follows:

- Population: patients or teeth that underwent orthodontic treatment.
- Intervention: orthodontic treatment with fixed appliances.
- Comparison: control group/teeth without orthodontic treatment.
- Outcome: amount/presence/absence of cervical root resorption.
- Study design: randomized clinical trials (RCTs), cohort studies, and case-control studies.

Any studies that fulfilled the following criteria were selected: (1) assessment of cervical root resorption as an outcome; (2) participants of any age or gender that had fixed appliances as an intervention; (3) participants with good general health and no systemic diseases; (4) participants with no previous history of trauma; (5) participants with no radiographic evidence of bone loss; (6) participants with periodontally sound teeth; and (7) limited to RCTs, cohort studies, and case-control studies.

Papers that had the following criteria were excluded: (1) pilot studies if the full RCT has been published, (2) studies with no full-text articles in English, (3) Studies performed on nonhumans, and (4) studies that included periodontally compromised or endodontically treated teeth for assessment.

### Search Strategy

A comprehensive search strategy was performed using electronic and manual search methods to locate indexed and nonindexed articles. Hand-searching of reference lists of included articles was also performed.

The electronic search strategy was done using a combination of medical subject headings (MeSH), nonmedical terms, and keywords. No limit on the date of publication was applied. The databases that were included in the electronic search strategy were PubMed, Wiley, ScienceDirect, and SCOPUS. The following keywords were used for this search method, adapted to each database, respectively.

(ortho* OR “tooth movement” OR “appliance”) AND cervical AND root AND resorption.
((ortho* OR “tooth movement” OR appliance) AND treatment OR “risk factor” OR induced) AND “cervical resorption.”
(orthodontic OR “tooth movement” OR “appliance”) AND “cervical resorption.”
(ortho* OR “tooth movement” OR “appliance” OR force) AND cervical AND root AND resorption.

The manual hand-search method included the following six journals:

1. *Journal of Orthodontics* (2000–2021).
2. *European Journal of Orthodontics* (2000–2021).
3. *American Journal of Orthodontics and Dentofacial Orthopedics* (2000–2021).
4. *Angle Orthodontist* (2000–2021).
5. *Journal of Endodontics* (2000–2021).
6. *Journal of Clinical Periodontology* (2000–2021).

The above-mentioned databases and hand-search were performed independently by two investigators (S.M. and T.M.). If disagreements arose, this was resolved through discussion. If no consensus was reached, a third author (K.K.) was then consulted for resolution.

### Risk of Bias Assessment and Quality of Evidence

All articles that were included in the study were reviewed independently by two authors for assessment of the level of bias. The Cochrane risk of bias tool (RoB 2) 15 was used to assess the risk of bias for RCTs, while the risk of bias in nonrandomized studies of interventions (ROBINS-I) 16 tool was used to assess the risk of bias of the other types of studies. The overall risk of bias of each study was allocated in accordance with the RoB 2 and ROBINS-I guidance handbooks. 17 18 An agreement was formed between the two assessors (S.M. and T.M.) prior to reaching a final decision regarding the overall risk of bias for each study. If any disagreement occurred, a third individual (K.K.) was consulted to reach a final decision.

Quality of evidence provided by this systematic review was assessed according to grading of recommendations, assessment, development, and evaluation (GRADE). 19 The overall quality of evidence was assessed based on five domains: risk of bias, inconsistency, imprecision, indirectness, and publication bias. An overall of very low, low, moderate, or high was then assigned according to the above-mentioned domains.

### Data Extraction and Data Analysis

Before extracting the data, a customized data extraction form was created to retrieve relevant information from each eligible study. The data extracted from the eligible studies included: participants' demographics, type of study, setting, intervention group, control group, primary outcome measure, secondary outcome measures, main findings of the study, and the follow-up period. This process of data extraction was performed independently by two reviewers (S.M. and T.M.). Any conflicts that occurred were resolved by a third reviewer (K.K.). A summary of this information can be seen in Tables 1 and 2 .

**Table 1:** Summary of characteristics of studies included in this review

	Dudic et al 18 (2017)	Giannopoulou et al 19 (2008)	Thönen et al 20 (2013)
Participant demographics	30 patients (20 females and 10 males) with a mean age of 16.7 years, scheduled to start orthodontic treatment that requires extractions of at least the two or four first or second premolars	16 patients (12 females and 4 males) with a mean age of 17.7 years, scheduled to start orthodontic treatment that requires extractions of at least the two or four first or second premolars	175 patients for final recall between November 2009 and March 2011 after brackets removal
Type of study	Randomized clinical trial	Randomized clinical trial	Retrospective cohort
Setting	Geneva University Hospital, Switzerland	Geneva University Hospital, Switzerland	University of Tampere, Tampere, Finland

**Table 2:** Summary of data from studies included in this review

	Dudic et al 18 (2017)	Giannopoulou et al 19 (2008)	Thönen et al 20 (2013)
Intervention group	59 premolars were randomly assigned to an experimental group. The premolars were then tipped buccally with 1 N force with a transpalatal and lingual arch as anchorage.	29 premolars were randomly assigned to an experimental group. The premolars were then tipped buccally with 1 N force with a trans-palatal and lingual arch as anchorage.	All patients that were treated with fixed appliances at the University clinic and had a final recall between November 2009 and March 2011.
Control group	58 premolars were bonded with brackets but were not moved orthodontically.	18 premolars were bonded with brackets but were not moved orthodontically.	Not applicable.
Primary outcome	To determine whether orthodontically induced tooth movement is correlated with the amount of cervical root resorption.	To assess the relationship between periodontal parameters and cervical root resorption in orthodontically moved teeth.	To identify the occurrence of cervical root resorption in molar teeth of orthodontic patients who were treated with fixed appliances.
Secondary outcome(s)	To determine whether the amount of cervical root resorption is the same in the maxilla and mandible. To determine whether the presence of an inter- or intraarch obstacle can reduce the amount of cervical root resorption.	To assess presence of cervical root resorption in orthodontically moved teeth.	Not applicable.
Main findings	There was a significant difference in mean cervical root resorption between both groups. The experimental group had 0.00055 mm ^3^ of cervical volume lost compared with the control group that had 0.00003 mm ^3^ of cervical volume loss.	There was a significant difference in cervical root resorption between both groups. Buccal cervical root resorption was detected in 27 out of the 29 experimental premolars (93%) and in 1 out of the 18 control premolars (5%).	No clinical signs of cervical root resorption were detected in any patient. Cervical root resorption was identified in one patient after checking with CBCT. No relationship was established between fixed appliances use and cervical root resorption.
Follow-up period	8 weeks	77 days	Average time period of a final recall after brackets removal was 8 ± 2 years

Abbreviation: CBCT, cone-beam computed tomography.

All data from the included studies would ideally be analyzed using a meta-analysis if the following conditions were met, that is, the included studies were homogeneous in regard to study design and outcome measures reported and were of low risk of bias. If those conditions were not met, a descriptive analysis would be performed instead.

## Results

### Selection of Studies

**Fig. 1:**
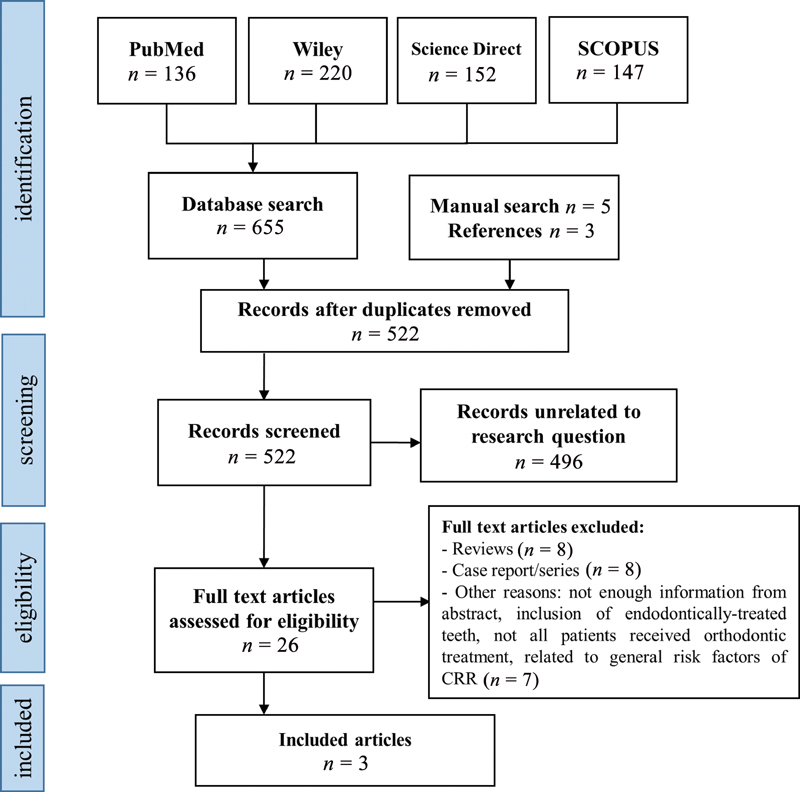
A flowchart that shows the process of identifying, screening, and selecting eligible studies using the Preferred Reporting Items for Systematic Reviews and Meta-Analysis (PRISMA) guidelines.

Fig. 1 contains a flow chart which identifies the included and excluded articles in each step. A total of 663 articles were assessed, including 655 articles from the electronic database search, 5 from the manual hand-search, and 3 from the reference lists. After removing all duplicates, 522 articles were left for screening, of which 496 were found to be unrelated to the research question, thus leaving 26 articles for potential inclusion in the study. After further inspection of the full texts of these articles, 23 were excluded for various reasons as follows: 8 were reviews; 8 were case reports or series; and 7 articles were excluded for other reasons including the lack of information from the abstract alone, the inclusion of endodontically treated teeth, not all patients receiving orthodontic treatment, or because the study was more focused on general risk factors of cervical root resorption rather than the risk of cervical root resorption in relation to an orthodontic treatment. This leaves three studies for inclusion, including two RCTs and one retrospective cohort study.

### Risk of Bias

**Fig. 2:**
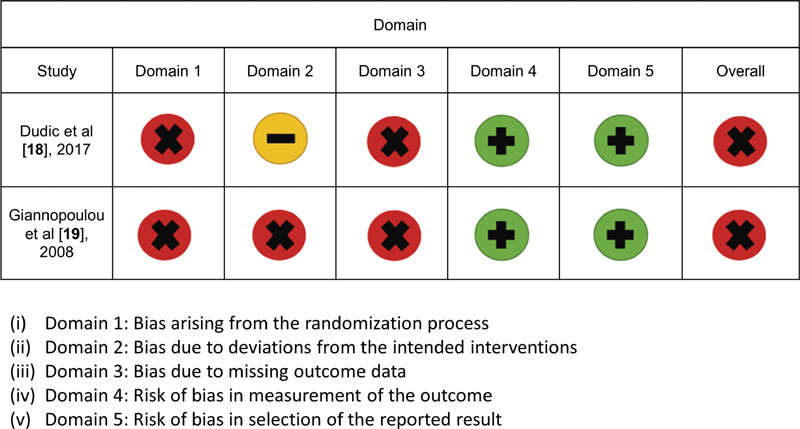
Risk of bias assessment results using the Cochrane risk of bias (RoB 2) tool for randomized clinical trial studies.

**Fig. 3:**
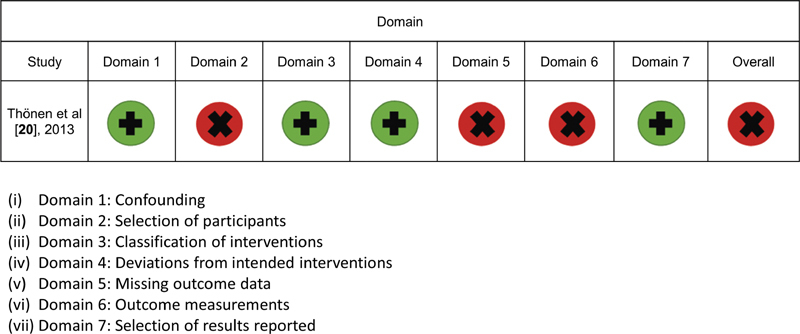
Risk of bias assessment results using the risk of bias in nonrandomized studies of interventions (ROBINS-I) tool.

Figs. 2 and 3 show a summary of the risk of bias judgment within each domain for the two RCTs and the one retrospective cohort study, respectively. Both RCTs 20 21 had an overall high risk of bias using the RoB 2 tool, whereas the cohort study 21 had an overall critical risk of bias in accordance with the ROBINS-I tool.

### Results of Individual Studies

Both RCTs 20 21 reported statistically significant differences ( *p* < 0.001) in cervical root resorption between orthodontically moved teeth and teeth that were not moved. Dudic et al 20 reported that after 8 weeks of applying a force of 1 Newton (N) buccally on 59 premolars in the experimental group, they exhibited a mean cervical root resorption of 0.00055 mm ^3^ (standard deviation [SD] = 0.00037) compared with a mean cervical resorption of 0.00003 mm ^3^ (SD = 0.00010) in the control group. The amount of cervical root resorption within the same individual was also significantly correlated ( *p* = 0.023, *R* = 0.421). It was also reported that there was a significant correlation (95% confidence interval [CI]: −0.00029, −0.00001, *p* = 0.045) between root resorption of the mandibular premolars (0.00062 mm ^3^ , SD = 0.00032 mm ^3^ ) and the maxillary premolars (0.00047 mm ^3^ , SD = 0.00042 mm ^3^ ). When comparing the experimental premolars to the control premolars, it was concluded that the amount of root resorption was significantly correlated with the amount of tooth displacement, with and without accounting for the presence of an obstacle ( *p* = 0.016, *R* = 0.318). Giannopoulou et al 21 reported that after applying a buccally directed force of 1 N on 29 premolars for 77 days, severe buccal cervical resorption was detected in 27 (93%) of the experimentally moved teeth in comparison to one (5%) of the 18 control premolars. Moderate resorption was seen on one (3.4%) premolar in the experimental group and six (33%) premolars in the control group. These differences were statistically significant ( *p* < 0.001).

The retrospective cohort study 22 aimed to identify the occurrence of cervical root resorption in the first and second molars of 175 invited patients during their final recall visit between November 2009 and March 2011. The average time after their brackets removal was 8 ± 2 years. It was found that none of the available 108 patients had clinical signs of cervical root resorption. One molar out of 858 (0.11%) had cervical root resorption, and three molars (0.35%) had surface resorption. The study concluded that there was no causative correlation between fixed orthodontic treatment and cervical root resorption.

### Quality of Evidence

The quality of evidence according to GRADE for all outcome measures was found to be of very low quality due to the following reasons: the high risk of bias of both RCTs and the critical risk of bias of the non-RCT study, all three studies had an inadequate sample size, short or variable follow-up times, and missing outcome data that were not taken in consideration when the data were analyzed.

## Discussion

This systematic review aimed to assess whether orthodontic treatment was a risk factor of cervical root resorption. After an extensive literature search, only three studies were eligible for inclusion in the review, of them two were RCTs 20 21 and one a retrospective cohort study. 22 Both RCTs reported a significant correlation between orthodontic treatment and cervical root resorption, whereas the cohort study reported no such an association.

The findings of the two RCTs included in this review agree with several previous studies that have suggested that orthodontic treatment, along with other factors, could be considered as a possible cause of cervical root resorption. 12 23 24 25 26

The use of bite-wing and periapical radiographs are often used to detect this pathological condition but this provides limited information on the identification of resorption lesions on root surfaces. Therefore, the use of cone-beam computed tomography (CBCT) is recommended instead, as it provides a higher three-dimensional (3D) diagnostic accuracy and vision. 27 28 29 Micro-CT has often been used and limited to research in dentistry at the present time. It has a significantly lower radiation dose when compared with conventional CTs and provides radiographs with excellent quality which can be analyzed through the use of 3D imaging software. 30 This micro-CT scanner along with a 3D imaging software was used in both RCTs included in this review 20 21 to assess the volume of cervical root resorption affecting the premolar teeth. The use of a micro-CT scanner may have contributed to the detection of the significant correlation between orthodontically moved teeth and cervical root resorption as it can give a rapid and accurate assessment without the need for clinical signs or routine radiographic signs for a diagnosis.

On the other hand, Thönen et al, 22 in their retrospective cohort study, used bite-wing radiographs that were checked by two investigators independently to determine whether there were any signs of cervical root resorption in first and second molars. If no clear conclusion was reached, a CBCT was used to confirm the diagnosis. While this method is considered safer compared with other methods from a radiation exposure perspective, some cases of cervical root resorption may have been underdiagnosed, especially those at the early stages of development. In contrast to the two RCTs, this cohort study did not consider surface-level resorption as a sign of cervical root resorption but instead described it as a self-limiting form of resorption. However, when taking Heithersay's classification 31 of cervical root resorption into consideration, the difference between class 1 lesions which involve superficial penetration into the dentin and surface-level cervical resorption may be minuscule and there is no guarantee that these surface-level lesions will remain self-limiting.

In the two RCTs included in this review, a standardized force of 1 N was applied buccally on premolars in the experimental group using a sectional arch wire (0.019 × 0.025 Titanium Molybdenum Alloy [TMA]) with a transpalatal or lingual arch as an anchorage unit compared with the control premolars which did not have any orthodontic forces applied to them. This was performed using a split-mouth design where one side of the dental arch was randomly assigned to an experimental group and the contralateral side to the control group. Split-mouth RCTs are ideal for our research question as they have the advantage of a potential increase in statistical power, due to each patient being their own control. 32 However, while standardized forces for initiating buccal tipping movement provides more control of confounding factors, such as amount and duration of tooth displacement, this does not reflect the reality in routine orthodontic practice where the force level varies from heavy to light throughout orthodontic treatment. 33

Interestingly, Dudic et al 20 further investigated the effect of the location of teeth within the maxilla or mandible and the presence of interarch and intraarch obstacles on cervical root resorption. They found that premolars in the mandible experienced more resorption than the maxillary premolars which may be explained by the difference in bone density between the mandible and maxilla. The posterior maxilla contains less-dense bone when compared with the posterior mandible. When moving teeth in bone of high density, more force is required to result in tooth movement which may induce root resorption and explain this outcome. However, it can be argued that the posterior maxilla contains bone of a highly vascular and less dense nature 34 which, theoretically, may lead to higher levels of inflammatory cells at the site of force application and therefore more resorption activities of the bony structures. Furthermore, it was concluded that the presence of an obstacle decreased tooth movement but regardless of its presence or absence in the jaw, the amount of root resorption was still correlated with the extent of tooth movement. This may be attributed to fact that the intra- and interarch obstacles were not of great severity to increase the forces required to induce tooth movement to a level beyond the threshold of causing root resorption. However, it is difficult to provide a specific and definitive explanation of this finding as the authors did not specify the type of intra- and interarch obstacles in their study. Further studies are needed to investigate differences in rates of cervical root resorption between teeth in the maxilla and mandible and between different levels of force applications to confirm the above findings.

The cohort study included in this review 22 assessed cervical root resorption of first and second molars in the final recall visit of patients after removal of their fixed appliances (an average of 8 ± 2 years). Patients were given a questionnaire to assess their general health and to rule out any confounding factors. Molars are often used as anchorage units in orthodontic treatment meaning that they resist unwanted displacement. 35 This could possibly explain the lack of cervical root resorption in the majority of cases of the cohort study included in this review. 22 In this study, the sole case of cervical root resorption occurred in a partially erupted second molar that was moved across a large distance to take the place of the adjacent extracted first molar. The greater amount of force that was applied on this tooth may have stimulated cervical root resorption to a point where it presented radiographic signs. This explanation is further supported by the fact that the same patient who presented with this resorption also had surface-level cervical resorption on three other molars that had also been moved over a large distance. There was no mention of whether the rest of the molars in this study were heavily displaced or whether there were other molars that had surface-level resorption.

## Limitations

Due to the heterogeneity of the data, the study design of the included studies and their high risk of bias, the findings could not be pooled into a meta-analysis. Currently, there is a lack of high-quality studies in the existing literature regarding the relationship between orthodontic treatment and cervical root resorption.

Both RCTs had an overall high risk of bias. No random allocation sequence was specified and no information on allocation concealment was given in both studies which makes the process of assigning intervention more susceptible to conscious or unconscious manipulation by the investigators, 36 particularly in the study published by Dudic et al 20 which showed confounding factors such as the location of the premolars and the presence of any obstacles in the jaw. Both studies also showed some concerns regarding missing outcome data that were not addressed in the study. At the beginning of both studies, it was stated that each patient would have two or four premolars assessed and have at least one premolar assigned to either the experimental or control group. However, both studies assigned a higher number of premolars to the experimental group than the number of the total premolars in the control group, indicating that there were missing or additional premolars that were not accounted for. Moreover, in one RCT, 20 one patient was dropped from the study due to a technical error in the micro-CT device where it was unable to quantify the volume of root resorption of the patient and subsequently no appropriate analysis method was implemented.

The cohort study had an overall critical risk of bias. Out of their 175 patients who were invited to the study, 67 (38%) dropped out for a variety of reasons, leaving 108 patients. No appropriate analysis was performed to overcome this high dropout rate which led to this domain obtaining a critical grade. Another limitation of the cohort study was the difference of time between the final recall and removal of the fixed appliances which was reported to be an average of 8 ± 2 years. This broad range may have led to a systematic error in regard to measuring the outcome as patients who removed their fixed appliances earlier would have had a higher chance of having cervical root resorption detected. These limitations may have led to a skew of the reported outcomes.

All three studies included in this review had small sample sizes which reduced the chance of detecting a true effect due to their low statistical power. Moreover, both RCTs had a short follow-up period. In one RCT, 20 force was applied on the selected premolars for 8 weeks, while the other RCT 21 allowed a time period of 77 days, with both time frames being too short to be applicable in the clinical setting. It should be noted that Giannopoulou et al 21 also reported that six premolars in the control group presented with moderate resorption compared with a one premolar in the experimental group. This may indicate the presence of a confounding factor that the authors did not account for which could be solved through the use of a questionnaire about potential risk factors of cervical root resorption. Another possible explanation of such a finding is the resorption could be an idiopathic in nature; however, due to the short follow-up period, any possible explanations would be speculative.

## Conclusion

Orthodontic treatment appears to have the potential of stimulating cervical root resorption; however, due to the lack of high-quality studies, the association between orthodontic treatment and cervical root resorption is still unclear. Patients undergoing fixed appliance therapy should be monitored and followed-up during and after the completion of their orthodontic treatment to detect any signs of cervical root resorption.
